# Incidence of multiple sclerosis among European Economic Area populations, 1985-2009: the framework for monitoring

**DOI:** 10.1186/1471-2377-13-58

**Published:** 2013-06-12

**Authors:** Enrique Alcalde-Cabero, Javier Almazán-Isla, Antonio García-Merino, Joao de Sá, Jesús de Pedro-Cuesta

**Affiliations:** 1 National Centre for Epidemiology, Carlos III Institute of Health, and Consortium for Biomedical Research in Neurodegenerative Diseases (CIBERNED), Av Monforte de Lemos 5, Madrid 28029, Spain; 2 Neurology Department, Puerta de Hierro Clinic, Madrid Autonomous University, Majadahonda, Spain; 3 Neurology Department, Santa Maria Hospital, Av. Prof. Egas Moniz, Lisbon 1600-001, Portugal

**Keywords:** Incidence, Monitoring, Multiple sclerosis, Time-trends, Surveillance

## Abstract

**Background:**

A debate surrounding multiple sclerosis epidemiology has centred on time-related incidence increases and the need of monitoring. The purpose of this study is to reassess multiple sclerosis incidence in the European Economic Area.

**Methods:**

We conducted a systematic review of literature from 1965 onwards and integrated elements of original research, including requested or completed data by surveys authors and specific analyses.

**Results:**

The review of 5323 documents yielded ten studies for age- and sex-specific analyses, and 21 studies for time-trend analysis of single data sets. After 1985, the incidence of multiple sclerosis ranged from 1.12 to 6.96 per 100,000 population, was higher in females, tripled with latitude, and doubled with study midpoint year. The north registered increasing trends from the 1960s and 1970s, with a historic drop in the Faroe Islands, and fairly stable data in the period 1980-2000; incidence rose in Italian and French populations in the period 1970-2000, in Evros (Greece) in the 1980s, and in the French West Indies in around 2000.

**Conclusions:**

We conclude that the increase in multiple sclerosis incidence is only apparent, and that it is not specific to women. Monitoring of multiple sclerosis incidence might be appropriate for the European Economic Area.

## Background

Multiple sclerosis (MS) is a chronic, demyelinating disease with frequent worsening episodes denoted as bouts or MS exacerbations, which are characteristic of the so-called relapsing-remitting form (RRMS) and absent in the less common, primary progressive form (PPMS). Despite intensive research over decades and early identification of susceptibility genes [1], its aetiology remains poorly known, with more than 50 susceptibility alleles identified and a considerable proportion of these regulated by Vitamin D [2,3]. Environmental factors, probably acting before adulthood, also appear to be implicated, as reflected by birth-cohort and season-of-birth effects [4,5], selected results of twin studies [6] and changing incidence among populations migrating to environments with different risk [7].

The most lively debate surrounding MS epidemiology at the beginning of the millennium [8-10] could perhaps be said to have centred on time-related increases in MS incidence (MSI), whether genuine or ascertainment-related. Subsequently and due, moreover, to potential post-vaccination incidents or side-effects of immunomodulating therapies, interest in MSI monitoring or surveillance gained wider recognition [11-15]. Several MS registers/studies have developed tools for, e.g., correcting the effects of diagnostic delays or using capture-recapture methods, appropriate for improving the quality and comparability of MSI measurements [16-18]. Design of incidence thresholds and other alarm signals may be problematic, since there is considerable uncertainty worldwide about expected MSI in specific populations for a number of reasons, such as rising trends, seasonality of bouts and validity of diagnostic criteria. The goals and formal components of public health-sponsored surveillance of MS have not yet been defined.

The purpose of this paper was twofold: firstly, it was an attempt to describe and compare MSI reported in recent decades among populations served by medical systems providing regular access to qualified neurological expertise, magnetic resonance imaging (MRI) and new treatments, potentially covered by publicly-run or -funded national health services, such as those of the European Economic Area (EEA) Member States. The non-EU members of EEA (Iceland, Liechtenstein and Norway) have agreed to enact legislation similar to that passed in the EU in the areas of social policy, consumer protection, environment and statistics. Secondly, the paper aimed to explore and describe the historical presence in the above-mentioned survey populations of three, specific, potentially interrelated MS-incidence features: 1) pseudo-periodic or occasional changes in incidence [19-21]; 2) changes in magnitude or shape of the age-specific incidence curve, occasionally reported as bimodal [20,22] and attributed to an increase in incidence among women aged over 40 years [10,23,24]; and, 3) a set of interrelated changes, perceived as a function of calendar time and interpreted as components of potential alarm signals (rising incidence of RRMS; increasing female/male incidence rate ratio and shortened diagnostic delay from clinical onset) [19,25-28].

## Methods

Principles for the undertaking of systematic reviews were followed [29].

### Study identification

We searched for reports in MEDLINE using “Multiple Sclerosis” and “Incidence”, both as MeSH terms and as TEXT WORDS issued between 1 January 1965 and 31 May 2012. The search yielded 5317 unrepeated documents. Analysis of document titles by two authors, EAC and JPC, searching for issues linked to MSI measurements, enabled identification by mutual agreement for the purposes of perusing the abstract (where available) of 344 of the above-mentioned 5317 documents plus a further six obtained from one of the author’s files. The same two authors then examined the abstracts, identified surveys reporting incidence periods from 1985 onwards, and mutually agreed on 122 of the above-mentioned 350 documents for full-text review. At a later step, the following two criteria sets were used for selection of papers for analysis of age- and sex-specific MSI from 1985 onwards, and time-related changes in MSI in EEA populations including those predating 1985.

#### Survey selection for studying age- and sex-specific MSI

The quality criteria used for results analyses were: (1) use of diagnostic criteria explicitly designed for MS/MS forms; (2) study incidence period either wholly post-1985 or, alternatively, at least two thirds post-1985 in cases where 1985 was included; (3) provision of age- and sex-specific measurements. Where different periods were covered by different reports for the same geographically-defined population, the most informative (usually the most recent) was selected. Exclusion criteria were as follows: (1) lack of reference to MS forms included in counts; (2) arbitrarily chosen accrual of ≤30 cases; and, (3) reports in languages other than Danish, English, French, Norwegian, Spanish or Swedish.

#### Survey selection for reviewing time-trends or time-related changes

The quality criteria applied were different and less strict than those used for age- and sex-specific incidence, and required that: 1) measurements included either crude or age- and sex-adjusted incidence rates for an observation period of, at least, an arbitrary 10-year duration in cases where 1985 was included; 2) explicit MS diagnostic criteria were used; 3) figures were based on clinical onset rather than MS diagnostic periods.

### Data-extraction or -completion

#### Study of age- and sex-specific MSI

**Figure 1:**
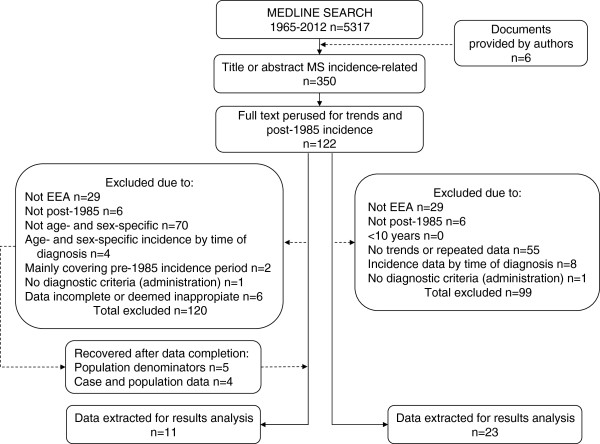
Attrition flow towards study selection.

A full-text review by both authors of a number of selected articles on age- and sex-specific MSI measurements in geographically-defined EEA populations suggested that there were insufficient or inadequate reported age-group data to be combined using models, due to one or more of the following factors: being represented only in graphs [30-32]; being incomplete for population groups, i.e., having numerators with zero cases generally in the youngest or oldest age-groups [8,33-37]; containing gross errors in rate calculations, i.e., for incidence in both sexes [38]; pertaining to age-groups that were too wide, selected (in general truncated) or mismatched [39-42]; or corresponding to protracted incidence periods, i.e., 1965-1993, 1968-1997 or 1975-1994, which probably encompassed multiple changes in MS diagnostic policies/traditions that were potentially heterogeneous by age at onset [43-45]. To complete the data, authors of reported surveys were thus contacted in specific instances by JPC via e-mail, correspondence address or telephone number of their institutional affiliations (usually hospital departments). Thus, for reports not yet definitely excluded on the basis of criteria defined in subsection 1.a. above, the authors of 13 surveys [30-35,37-42,46] were requested to furnish such data in a different format, e.g., age at clinical onset instead of age at MS diagnosis, or distribution by age of incident MS case different to that shown. Subsequently, new case-related data were obtained from five surveys [30-32,37,40] or completed with data extracted from graphs, specifically from four of them deemed useful for combination [31,32,37,40]. For 12 surveys, the population structure in new age-groups or complementary demographic data were obtained from the authors or official statistics bodies [8,30-37,40,46,47]. Eight of the 12 surveys for which new data were requested/obtained [8,31,32,34-37,40] fulfilled criteria for inclusion of study results. Since the data from Modena were included in two different reports [36,47], we studied a total of ten age-and sex-specific incidence sets drawn from nine different populations [8,10,31,32,34-37,40,48]. Only two of the eleven surveys finally selected for study provided reported data initially deemed valid for analysis purposes [10,48] (see Figure 1 for a summary description of attrition flow). Reported or obtained numerators and denominators used for analyses are presented in the Table 1.

**Table 1:** **Reported and rearranged data from MS incidence surveys presented in Figure**3

**Study**		**Men**			**Women**			**Both**		
**First author Reference**	**Age group**	**Cases**	**Person-years**	**Rate**	**Cases**	**Person-years**	**Rate**	**Cases**	**Person-years**	**Rate**
Debouverie 2007 [31]	0-14	0	3,070,347	0.00	7	2,911,480	0.24	7	5,981,827	0.12
	15-24	59	2,191,949	2.69	213	2,072,309	10.28	272	4,264,258	6.38
	25-34	154	2,236,211	6.89	445	2,199,900	20.23	599	4,436,111	13.50
	35-44	151	2,262,937	6.67	339	2,249,492	15.07	490	4,512,429	10.86
	45-54	93	1,827,995	5.09	137	1,782,369	7.69	230	3,610,364	6.37
	55-64	21	1,458,543	1.44	32	1,542,168	2.08	53	3,000,711	1.77
	65+	2	1,680,520	0.12	5	2,584,219	0.19	7	4,264,739	0.16
Guidetti 1995 [36]	0-14	0	449,088	0.00	0	428,838	0.00	0	877,926	0.00
	15-24	12	437,867	2.74	20	424,286	4.71	32	862,153	3.71
	25-34	7	429,913	1.63	25	425,059	5.88	32	854,972	3.74
	35-44	9	420,283	2.14	16	419,570	3.81	25	839,853	2.98
	45-54	1	407,265	0.25	11	409,216	2.69	12	816,481	1.47
	55-64	0	384,447	0.00	2	416,345	0.48	2	800,792	0.25
	65+	0	414,027	0.00	0	595,139	0.00	0	1,009,166	0.00
Alonso 2007 [48]	0-14	0	1,114,646	0.00	0	1,061,240	0.00	0	2,175,886	0.00
	15-24	6	711,643	0.84	24	700,702	3.43	30	1,412,345	2.12
	25-34	50	928,136	5.39	115	926,436	12.41	165	1,854,572	8.90
	35-44	57	860,894	6.62	155	837,502	18.51	212	1,698,396	12.48
	45-54	51	800,963	6.37	111	780,853	14.22	162	1,581,816	10.24
	55-64	29	592,600	4.89	31	596,522	5.20	60	1,189,122	5.05
	65+	5	754,786	0.66	8	1,078,718	0.74	13	1,833,504	0.71
Nicoletti 2005 [32]	0-14	2	297,120	0.67	1	281,850	0.35	3	578,970	0.52
	15-24	10	251,490	3.98	19	246,740	7.70	29	498,230	5.82
	25-34	22	237,360	9.27	34	240,670	14.13	56	478,030	11.71
	35-44	12	205,920	5.83	25	226,150	11.05	37	432,070	8.56
	45-54	7	177,620	3.94	17	203,130	8.37	24	380,750	6.30
	55-64	3	158,980	1.89	3	193,760	1.55	6	352,740	1.70
	65+	0	205,740	0.00	0	295,230	0.00	0	500,970	0.00
Joensen 2010 [40]	0-14	0	125,615	0.00	0	118,653	0.00	0	244,268	0.00
	15-24	3	81,263	3.69	6	69,516	8.63	9	150,779	5.97
	25-34	5	74,267	6.73	9	63,934	14.08	14	138,201	10.13
	35-44	6	75,305	7.97	5	66,389	7.53	11	141,694	7.76
	45-54	3	64,103	4.68	5	55,218	9.06	8	119,321	6.70
	55-64	0	49,604	0.00	1	44,487	2.25	1	94,091	1.06
	65+	0	59,298	0.00	1	71,896	1.39	1	131,194	0.76
Sundstrom 2003 [37]	0-14	1	255,257	0.40	1	244,079	0.40	2	499,336	0.40
	15-24	5	175,802	2.90	14	169,431	8.40	19	345,233	5.60
	25-34	18	179,111	10.00	32	166,142	19.20	50	345,253	14.40
	35-44	16	182,644	8.70	23	171,520	13.40	39	354,164	10.90
	45-54	7	163,908	4.20	12	157,093	7.50	19	321,001	5.90
	55-64	0	126,402	0.00	4	131,128	3.10	4	257,530	1.60
	65+	0	189,679	0.00	0	239,325	0.00	0	429,004	0.00
Granieri 2007 [34]	0-14	0	247,388	0.00	2	232,900	0.86	2	480,288	0.42
	15-24	10	282,723	3.54	17	267,370	6.36	27	550,093	4.91
	25-34	21	370,041	5.68	55	353,479	15.56	76	723,520	10.50
	35-44	13	348,169	3.73	41	349,768	11.72	54	697,937	7.74
	45-54	8	336,728	2.38	18	350,939	5.13	26	687,667	3.78
	55-64	1	331,387	0.30	4	366,978	1.09	5	698,365	0.72
	65+	0	443,139	0.00	2	656,512	0.30	2	1,099,651	0.18
Granieri 2008 [35]	0-14	0	31,856	0.00	0	28,859	0.00	0	60,708	0.00
	15-24	2	25,489	7.85	4	24,735	16.17	6	50,196	11.95
	25-34	3	35,513	8.45	11	40,625	27.08	14	76,022	18.42
	35-44	0	32,290	0.00	5	33,221	15.05	5	65,509	7.63
	45-54	2	27,336	7.32	2	27,582	7.25	4	54,887	7.29
	55-64	1	22,396	4.47	2	22,356	8.95	3	44,736	6.71
	65+	0	27,157	0.00	1	36,490	2.74	1	63,483	1.58
Nicoletti 2010 [10]	0-14	0	129,630	0.00	0	122,860	0.00	0	252,490	0.00
	15-24	3	103,300	2.90	13	101,785	12.77	16	205,085	7.80
	25-34	10	115,110	8.69	27	114,755	23.53	37	229,865	16.10
	35-44	11	104,500	10.53	20	114,380	17.49	31	219,880	14.10
	45-54	11	98,320	11.19	8	105,025	7.62	19	198,345	9.58
	55-64	4	76,320	5.24	1	91,740	1.09	5	168,060	2.98
	65+	0	111,475	0.00	0	167,980	0.00	0	275,855	0.00
Cabre 2009 [8]	0-14	1	1,388,049	0.07	2	1,351,144	0.15	3	2,739,193	0.11
	15-24	6	848,215	0.71	17	837,479	2.03	23	1,685,694	1.36
	25-34	8	808,481	0.99	35	926,145	3.78	43	1,734,626	2.48
	35-44	7	814,933	0.86	30	931,847	3.22	37	1,746,780	2.12
	45-54	2	608,781	0.33	18	699,425	2.57	20	1,308,206	1.53
	55-64	1	447,677	0.22	2	511,278	0.39	3	958,955	0.31
	65+	0	530,373	0.00	1	731,100	0.14	1	1,261,473	0.08

#### Review of time trends or time-related changes

No data were requested from authors for this purpose. Review results for specific MSI, generally at 5- or, alternatively, at >5- [8,10,19,21,27,28,34,36], [41,47,49-56] or 1-year calendar time intervals [31,57,58] were obtained from original reports, disregarding other study-interval durations. Updated data were used for Ferrara [34,43] and Catania [10,32].

### Incidence measurements and comparisons

#### Rates and specific indicators

For each specific survey, age- and sex-adjusted incidences were computed using the European standard population. Female/male (F/M) ratios were obtained, firstly from the number of cases and secondly from stratified analysis (Mantel-Haenszel estimator i.e., F/M M-H). Where not reported, diagnostic delay was calculated from differences between mean or median ages at onset and diagnosis for either incidence or diagnostic periods, if mentioned.

#### Comparative measurements

Incidence rate ratios were obtained from log-linear models using the binomial distribution. Five analyses were performed using Poisson models. The core analysis was conducted by fitting a global model, in which the independent variables were age-sex, midpoint of study period and categorised diagnostic criteria. F/M ratios were computed from models. Time and latitude trends were described from linear change. Secondary analyses were conducted separately for populations of: both sexes aged below 35 years at onset; both sexes aged ≥35 years at onset; and women or men aged ≥35 years at onset. Calculations were made using the Stata 11 software package. Diagnostic criteria were categorised into the following three variable values: Poser clinically definite and probable; Poser and McDonald or other MRI dependent criteria; and other criteria (McAlpine, Schumacher, and mixed non-MRI related). Midpoint of study period for incidence, latitude in degrees, and F/M M-H obtained from prior analysis were used as continuous variables.

#### Time-trends

Two independent approaches to time-trends were adopted. Firstly, time-trends from 1985 onwards were studied using mid time points for survey data from models based on ten data sets. Secondly, the reported time series from incidence surveys providing either crude, or age- or age- and sex-adjusted figures for periods encompassing 1985 or after in the EEA were identified for 19 EEA populations in 21 reports. Trends were visually examined from crude or adjusted rates plotted in graphs. When providing the data requested, survey authors occasionally gave their views on the impact had by changes in patient-management traditions on changes in incidence: these views will be referred to below as personal communications.

## Results

**Figure 2:**
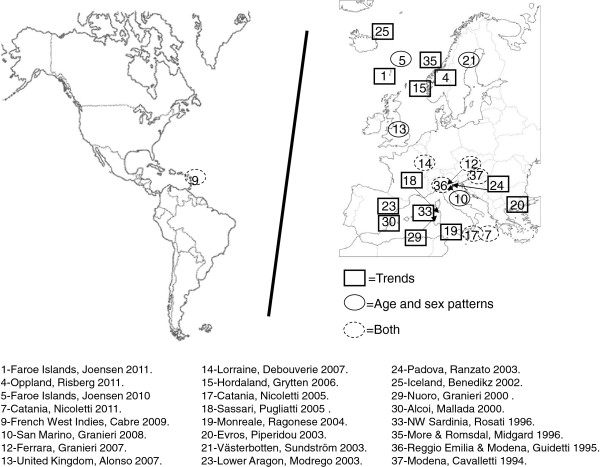
Geographical distribution of MS surveys included in age-specific incidence analysis (top) and time trends (bottom).

The above-mentioned 122 documents selected for full-text perusal generated the 37 articles listed in Table 2 and initially considered for potential data requests. Their epidemiological features prior to data completion are shown here mainly for the purposes of assessing possible selection bias and impact of omissions. In general, reported incidences referred to all MS forms, with two surveys providing separate, crude or age- and sex-specific RRMS and PPMS data [54,59]. Surveys reporting incidence for intervals prior to 1985 covered long study periods dating back several decades, e.g., up to 1943 for the Faroes [19,21] and 1955 for Nuoro, Italy [27]. We also included a survey conducted in San Marino, a mini-state not formally belonging to the EEA, with human development indicators similar to or higher than those of EEA countries and specialised medical services shared with Italy [35]. Of the 37 reports, four were used for comparing age-specific incidence only [35,37,40,48], 16 provided data for studying trends [19,21,27,28,41,43,49-58] and seven could be used for both purposes [8,10,31,32,34,36,47]. Ten studies were excluded [24,26,30,33,38,39,42,46], [59,60], four due to incidences calculated from data sets based on age at diagnosis, i.e., explicitly reporting MS diagnoses instead of clinical onsets [30,33,46,60]. The geographical distribution of studies selected for each purpose is depicted in Figure 2. Given the political concept of the EEA, surveys included ethnically, racially and geographically heterogeneous populations.

**Table 2:** Epidemiological features of selected studies

	**Study population**	**Study period**	**Study**	**Sex- and age-specific counts**	**Diagnostic criteria**	**Other relevant information**	**Data obtained from author or external sources (yes/no)**	**F/M ratio (incident cases)**	**Criteria fulfilled for inclusion in incidence, by age and sex, trend, both or none**
	**First author and reference**	**Number of years and intervals**	**Number of cases (period)**			**Diagnostic delay (DD) in years**			
						**Mean age at onset/diagnosis**			
1	Faroe Islands	1943-2007	81	NO	<1986 Poser CD,CPr	Mean age at onset	NO	1.25	Trend
	Denmark	65			>1986 Poser,	32y			
	Joensen 2011 [19]	7			McDonald and Thompson				
2	South-west Sardinia	1958-2007		YES	McDonald	-	NO		None
	Italy	50							
	Cocco 2011 [33]	5							
3	Iceland	2002-2007	136	YES	Poser CD for	Mean age at diagnosis 36.3y	YES	3	None
	Eliasdottir 2011 [46]	6			PPMS	Mean age at onset 32y			
		1							
4	Oppland	1989-2001	148	NO	Poser D and Pr, otherwise NS	-	NO	2.02	Trend
	Norway	13							
	Risberg 2011 [55]	3							
5	Faroe Islands	1986-2007	43	YES	Poser and/or McDonald, for PPMS	36y at onset	NO	1.53	Age- and sex-specific incidence
	Denmark	22							
	Joensen 2010 [40]	1							
6	Ostrobothnia	1992-2007	374	NO	Poser CD	Mean age at onset 31.7y	NO		None
	Finland	16			McDonald CD	Mean age at diagnosis 35.9y			
	Krokki 2010 [24]	1							
7	Catania	1975-2004	367 (75-04)	YES	Poser CD,Cpr,LSPr	Mean DD 1.4y	NO	1.55	Both
	Italy	30	108 (00-04)			Age at onset increasing with time			
	Nicoletti 2011 [10]	6							
8	Cardiff (Wales)	1985-2007	582	NO	Poser and McDonald	-	NO	2.83	None
	United Kingdom	23			(otherwise NS)				
	Hirst 2009 [38]	1							
9	West Indies	1992-2007	130	YES	Revised McDonald	Mean age at onset 34.2y	YES	4.2	Both
	France	15							
	Cabre 2009 [8]	3							
10	San Marino	1990-2005	33	YES	Poser D,Pr	Mean DD decreasing with time (1.5 to 0.08y)	YES	3.12	Age- and sex-specific incidence
	Italy	16							
	Granieri 2008 [35]	1							
11	Greece 3 regions	1984-2006	834	NO	Poser and McDonald D	Mean DD 2.62y decreasing with time (3.23 to 1.79y)	NO	1.38	None
	Papathanasopoulos 2008 [60]	23			(otherwise NS)	Mean age at onset 31.41y			
		4							
12	Ferrara	1965-2003	200 (90-03)	YES	Poser CD,CP	Mean DD decreasing with time (1.25 to 0.5y)	YES	2.21	Both
	Italy	39	421 (65-03)						
	Granieri 2007 [34]	4							
13	UK cohort	1993-2000	642	YES	Poser D,Pr,	-	NO	2.24	Age- and sex-specific incidence
	Alonso 2007 [48]	8			McDonald D,Pr				
		1							
14	Lorraine	1990-2002	1658	YES	Poser D,Pr	-	YES	2.45	Both
	France	13							
	Debouverie 2007 [31]	1							
15	Hordaland	1953-2002	878	NO	Poser	Mean DD decreasing with time (9.8-0.9y)	NO	1.9	Trend
	Norway	50							
	Grytten 2006 [51]	10							
16	Nordland	1970-1999	259 (70-99)	YES	Poser	Mean DD 4.7y	YES	1.87	None
	Norway	30	183 (85-99)		CDPr,,LSDPr	Mean age at diagnosis 39y			
	Alstadhaug 2005 [30]	6							
17	Catania	1975-1999	155 (90-99)	YES	Poser CDPr,LSDPr	Mean at onset 33.3y	YES	1.77	Both
	Italy	25				Mean DD 1.7y			
	Nicoletti 2005 [32]	5							
18	Sassari	1965-1999	689	NO	Poser	DD range 13.0-0.9y decreasing with time	NO	2.57	Trend
	Italy	34				Age at onset increasing with time			
	Pugliatti 2005 [54]	7							
19	Monreale City	1981-2000	19	YES	Poser D,Pr	Mean DD 9.2y	NO	-	Trend
	Italy	20							
	Ragonese 2004 [41]	2							
20	Evros	1974-1999	56	NO	Poser CD,LSD	-	NO	2.8	Trend
	Greece	26							
	Piperidou 2003 [53]	5							
21	Vasterbotten County	1988-1997	133	YES	Poser	-	NO	1.83	Age- and sex-specific incidence
	Sweden	10							
	Sundström 2003 [37]	1							
22	Finland 3 districts	1979-1993	1066	NO	Poser CD or Lublin et al for PPMS	Mean age at diagnosis for districts (35.7,39.3,39.7y)	NO	1.6	None
	Sumelahti 2003 [59]	15							
		2							
23	Lower (*Bajo*) Aragon	1985-2002	42	NO	Poser CD,CPr	DD 2y	NO	1.93	Trend
	Spain	18				Mean age at onset 29y			
	Modrego 2003 [58]	1							
24	Padova	1980-2000	580	NO	Poser	Decreasing DD with time	NO	1.92	Trend
	Italy	20				34y			
	Ranzato 2003 [28]	4							
25	Iceland	1951-1999	372	NO	Poser	-	NO	1.9	Trend
	Benedikz 2002 [49]	49							
		10							
26	Faroe Islands	1943-1994	54	NO	Poser CD,CPr	Individual age at onset available	NO	-	Trend
	Denmark	52							
	Kurtzke 2001 [21]	8							
27	Enna, Sicily	1986-1995	16	YES	Poser	Mean DD 3y	NO	1.28	None
	Italy	10							
	Grimaldi 2001 [39]	1							
28	Oslo	1972-1996	794	NO	Poser CD	For persons diagnosed 1986-1999	NO	2.38	None
	Norway	25				DD 5.2y			
	Celius 2001 [26]	5				Age at diagnosis 38.1y			
29	Nuoro	1955-1995	469	YES	Poser (unspecified), Allison & Millar and Schumacher	Mean DD 4.61y	NO	1.95	Trend
	Italy	41				Mean age at onset 28.5y			
	Granieri 2000 [27]	8							
30	Alcoi	1986-1997	45	NO	Poser CD,CPr	-	NO	4	Trend
	Spain	12				Mean age at onset 35.1y			
	Mallada 2000 [57]	1							
31	Troms & Finnmark	1974-1992	139	NO	Rose DPrPs &	Mean DD 4.5y	NO	1.36	Trend
	Norway	19			Poser CD,CPr,LSD	Mean age at onset/diagnosis available			
	Gronlie 2000 [50]	4							
32	Bagueria city	1985-1994	20	YES	Poser	Age at diagnosis 34.6y	NO	1.86	None
	Italy	10				Mean DD 2.6y			
	Salemi 2000 [42]	1							
33	North-western Sardinia	1962-1991	277	YES	Poser CD,CPr,LSD,LSPr	DD range 8.0-1.8y decreasing with time	NO	2.46	Trend
	Italy	30				Mean age at onset 27y			
	Rosati 1996 [56]	6							
34	Ferrara	1965-1993	252	YES	Poser CD,CPr	DD range 6.1-1.9y decreasing with time	NO	2.07	Trend
	Italy	29				Mean age at onset for specific forms			
	Granieri 1996 [43]	6							
35	More & Romsdal	1950-1991	330	NO	McAlpine	DD potentially available 6y	NO	1.41	Trend
	Norway	42				39.2y			
	Midgard 1996 [52]	8							
36	Reggio Emilia & Modena	1970-1990	316 (70-90)	YES	McAlpine CD,CPr	-	YES	2.01	Both
	Italy	6	105 (85-90)			Mean age at onset 30.75y			
	Guidetti 1995 [36]	4							
37	Modena	1970-1990	183 (70-90)	YES	McAlpine D,Pr	-	YES	1.9	Both
	Italy	6	59 (85-90)			Mean age at onset 30.8y			
	Cavalletti 1994 [47]	4							

Crude and age- and sex- adjusted incidence figures using the European standard population, data on diagnostic delay, latitude and mid time point, F/M M-H, and details of diagnostic criteria for the ten best-quality surveys are listed in Table 3. Effects of adjustment on incidence figures were modest, with crude figures for both sexes ranging from 1.14 to 7.93 per 100,000 person years, and age- and sex-adjusted figures ranging from 1.12 to 7.25 per 100,000 person years, with the extreme levels for the French West Indies and San Marino displaying an almost similar 6- to 7-fold variation. The highest incidence was reported for San Marino (1990-2005), with Poser definite and probable MS diagnostic criteria, and the lowest for the French West Indies (1992-2007), with McDonald criteria. F/M ratios calculated from case numbers ranged from 1.59 to 4.20, and those for F/M M-H had a similar range, 1.68 to 3.79, with figures being lower in the Faroes (1986-2007) and Catania (1990-1999) and higher in the French West Indies. The lowest incidence and highest F/M ratios were seen for the French West Indies. Latitudes varied continentally from 37.3 to 65.0 degrees north, with a 15.2 degree outlier in the French West Indies. The length of diagnostic delay from onset was reported in four surveys, all Italian, ranging from a mean of 21 months in Catania during the period 1990-1999, to a mean of 6.1 months in Ferrara in 2003 and one month (number of patients nor specified) in San Marino in 2005. The more recent the study period, the shorter the diagnostic delay. Poser diagnostic criteria were used in six surveys [10,31,32,34,35,37], McDonald or Poser/McDonald criteria combined in three [8,40,48], and McAlpine’s criteria in the first survey [36]. An aspect that remained unclear was the likelihood of laboratory-supported forms being included in Poser’s forms, denoted by authors as “definite and probable” [8,31,34,35,48], with the proportion of the laboratory-supported probable cases, where specified, being 11% [37]. The F/M ratio did not change with study midpoint using linear regression but showed a modest, albeit statistically significant, decrease when the French West Indies survey was excluded, -2.6% (-4.7%- -0.6%) annually. The F/M ratio decreased significantly with latitude, -2.9% (-3.5%- -2.2%) per degree, and modestly -0.6% (-1.4%- 0.2%) when French West Indian data were excluded (Table 3). Data were deemed too sparse for testing linear associations between incidences and length of diagnostic delays reported in Table 3 (data not shown), or between incidences and F/M figures.

**Table 3:** Selected data from surveys with available age- and sex-specific incidence for periods of clinical onset in EEA populations

**Survey population**	**No. cases**	**Diagnostic criteria**	**Incidence**			**Stratified**	**F/M**	**Diagnostic delay**	**Mid time point**	**Latitude**
**Incidence period**			**Crude**			**F/M M-H**	**ratio**	**Months**	**Year**	**Degrees N**
**Person/years**			**Adjusted**					**Mean/median DD**	**(1987-2000)**	
**Reference**			**Women**	**Men**	**Both**					
Lorraine	1658	Poser D,Pr, (NSO)	7.68	3.26	5.51	2.50	2.45	-	1996	49
1990-2002			7.76	3.16	5.43	(2.24-2.77)				
30070439										
Debouverie 2007 [31]										
Reggio Emilia & Modena	105	Mc Alpine D,Pr (NSO)	2.37	0.99	1.70	2.58	2.55	-	1987.5	44.28
1985-1990			2.45	0.95	1.69	(1.68-3.97)				
6061343										
Guidetti 1995 [36]										
United Kingdom	642	Poser D,Pr or MacDonald D,Pr (NSO)	7.42	3.44	5.47	2.26	2.24	-	1996.5	53.51
1993-2000			7.45	3.30	5.36	(1.91-2.67)				
11745641										
Alonso 2007 [48]										
Catania	155	Poser CDPr,LSDPr	5.87	3.65	4.81	1.68	1.77	21 m	1994.5	37.3
1990-1999			6.02	3.58	4.84	(1.21-2.33)				
3221760										
Nicoletti 2005 [32]										
Faroe Islands	43	Poser CD, LSD, or McDonald for one attack (separate criteria for PPMS forms)	5.51	5.21	4.32	1.82	1.59	-	1996.5	62.01
1986-2007			5.90	5.23	4.48	(0.99-3.35)				
1019548										
Joensen 2010 [40]										
Västerbotten	133	Poser CDPr, LSDPr	6.73	3.69	5.21	1.94	1.83	-	1992.5	65.02
1988-1997			7.23	3.72	5.43	(1.36-2.77)				
2551521										
Sundström 2003 [37]										
Ferrara	200	Poser D,Pr (NSO)	5.39	2.25	3.83	2.66	2.62	15.4 m (in 1990)	1996.5	44.5
1990-2003			5.77	2.18	3.96	(1.94-3.63)		6.1 m (in 2003)		
4937521										
Granieri 2007 [34]										
San Marino	33	Poser D,Pr (NSO)	11.69	3.96	7.93	2.95	3.13	18.3 m (in 1990)	1997.5	43.56
1990-2005			10.46	3.80	7.25	(1.32-6.56)		1 m (in 2005)		
415540										
Granieri 2008 [35]										
Catania	108	Poser CDPr,LSDPr	8.43	5.28	6.94	1.69	1.77	17 m	2002	37.3
2000-2004			8.72	5.24	6.96	(1.14-2.49)				
1549580										
Nicoletti 2011 [10]										
French West Indies	130	2005 revised McDonald (NSO)	1.75	0.46	1.14	3.79	4.20	-	2000	15.2
1992-2007			1.71	0.44	1.12	(2.45-5.87)				
11434927										
Cabre 2009 [8]										
Beta for F/M M-H in ordinates linear regression	-	-	-	-	-	-	-	-		
All data sets									0.022	-0.029
									(-0.006, 0.05)	(-0.035, -0.022)
(French West Indies excluded)									-0.026	-0.006
									(-0.047, -0.006)	(-0.014, 0.002)

**Figure 3:**
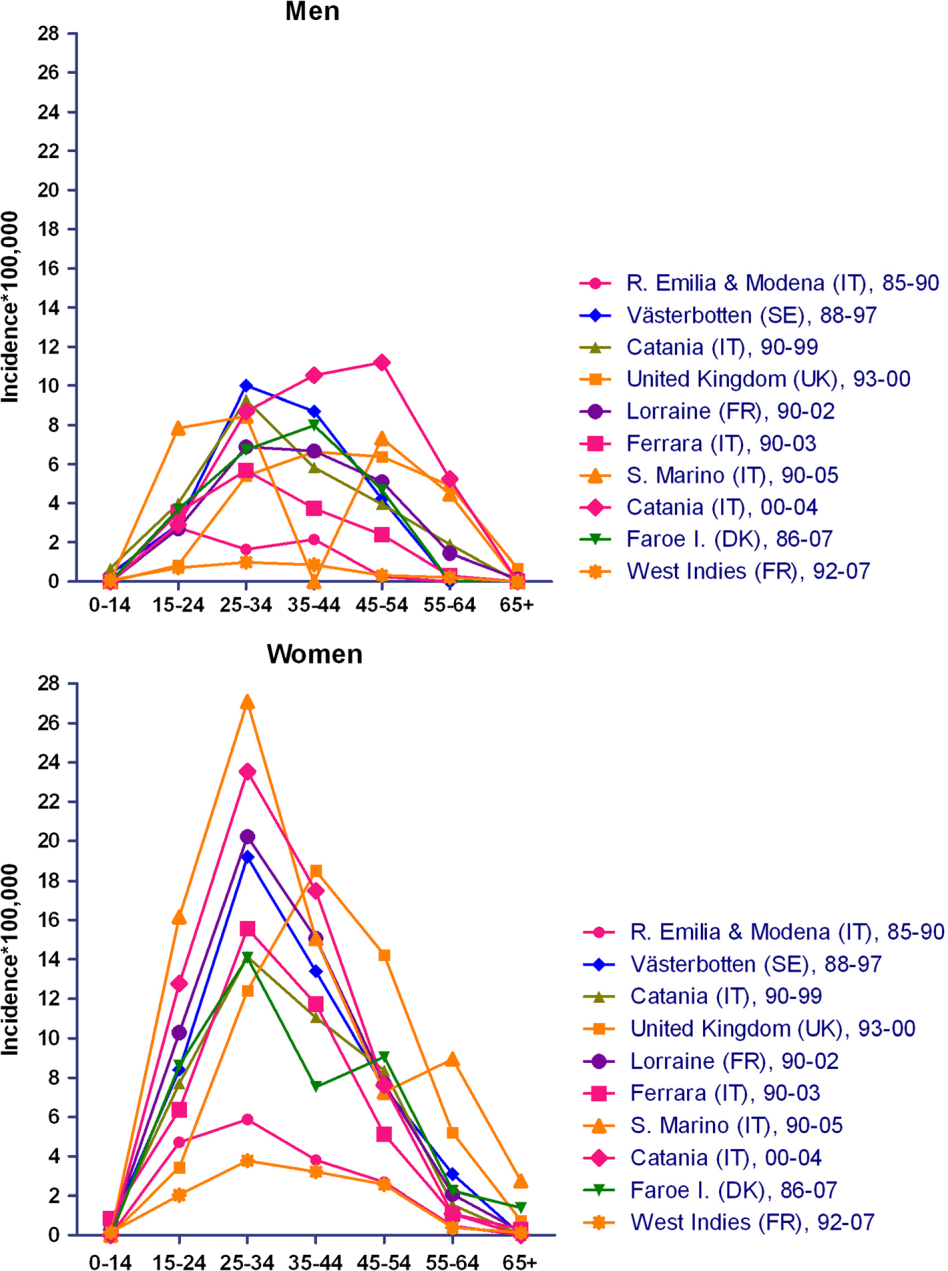
Age-and sex-specific incidences of selected EEA surveys.

Age- and sex-specific incidences for ten surveys are depicted in Figure 3. In general, incidence among women was nil at ages under 15 (five surveys) and ≥65 years, and low at ages 55-64 years, peaked at 25-34 years in general and at 35-44 years solely in the UK study, frequently reaching values of 20-30 per 100,000. The lowest figures were seen: for the survey conducted in Reggio Emilia-Modena in 1985-1990, using Poser criteria; and in particular, for the recent study conducted in the French West Indies in 1992-2007 using McDonald’s revised criteria. Among men, age-specific incidence was less stable and lower, suggesting a trend to peak at later ages in some recent surveys in the UK and Catania [10,48].

Results from log-linear models are described in Table 4. We used ten data sets from surveys undertaken in different, independent populations, fulfilling selection criteria for the study of age-and sex-specific incidence. Lorraine, a large and well-conducted study, was chosen as reference, and ages 25-34 and 35-44 years at onset were the reference for comparisons when analyses were restricted to ≤34 and ≥35 years respectively. Results were in general similar for the core analysis, women, men, ages below 35 years at onset and ages >35 years at onset, with negligible differences. As seen from the core model, 7-fold differences in RRs were seen, with differences in RRs in Catania, 1.39 (1.01-1.91), Reggio Emilia-Modena, 0.33 (0.24-0.45), and the French West Indies, 0.20 (0.16-0.29) proving statistically significant. Incidence among women was triple that of men 2.9 (1.87-2.57). The mean increase in incidence with time was 9% (4%-15%) per year in both sexes. Incidence was different when measured using MRI-based criteria (McDonald criteria included) in the core model, RR 0.68 (0.55-0.85).

**Table 4:** Results from log-linear models (binomial function), OR and 95%CI

		**Core analysis**	**Both sexes**	**Both sexes**	**Women**	**Men**
**Study population, incidence period**		**All ages both sexes**	**Age at onset <35y**	**Age at onset ≥35y**	**Age at onset ≥35y**	**Age at onset ≥35y**
First author and reference	Lorraine 1990-2002	1	1	1	1	1
	Debouverie 2007 [31]					
	Reggio Emilia 85-1989	0.33	0.37	0.25	0.28	0.18
	Guidetti 1995 [36]	(0.24-0.45)	(0.29-0.48)	(0.17-0.36)	(0.19-0.42)	(0.09-0.37)
	UK cohort 1993-2000	1.22	0.57	1.66	1.59	1.55
	Alonso 2007 [48]	(0.94-1.59)	(0.48-0.66)	(1.30-2.13)	(1.28-1.96)	(1.03-2.35)
	Catania 1990-1999	0.98	0.90	0.87	0.84	0.90
	Nicoletti 2005 [32]	(0.72-1.32)	(0.72-1.12)	(0.63-1.20)	(0.61-1.17)	0.53-1.50)
	Faroe Islands 86-2007	0.94	0.83	0.91	0.80	1.02
	Joensen 2011 [19]	(0.63-1.39)	(0.55-1.25)	(0.57-1.48)	(0.45-1.45)	(0.49-2.10)
	Västerbotten 88-1997	1.07	1.03	1.00	0.96	1.02
	Sundström 2003 [37]	(0.79-1.46)	(0.81-1.32)	(0.72-1.39)	(0.68-1.36)	(0.61-1.70)
	Ferrara 90-2003	0.74	0.79	0.64	0.74	0.49
	Granieri 2007 [34]	(0.55-0.99)	(0.65-0.97)	(0.48-0.86)	(0.55-0.98)	(0.29-0.81)
	San Marino 90-2005	1.44	1.43	1.19	1.34	0.81
	Granieri 2008 [35]	(0.93-2.23)	(0.92-2.23)	(0.66-2.15)	(0.71-2.54)	(0.25-2.61)
	Catania 2000-2004	1.39	1.19	1.43	1.06	2.07
	Nicoletti 2011 [10]	(1.01-1.91)	(0.90-1.57)	(1.01-2.00)	(0.72-1.57)	(1.26-3.38)
	West Indies 1992-2007	0.21	0.19	0.21	0.26	0.11
	Cabre 2009 [8]	(0.16-0.29)	(0.15-0.25)	(0.15-0.29)	(0.19-0.35)	(0.06-0.22)
Age-groups	0-14y	0.01	0.01	-	-	-
		(0.008-0.02)	(0.008-0.02)			
	15-24y	0.50	0.47	-	-	-
		(0.39-0.64)	(0.42-0.52)			
	25-34y	1	1	-	-	-
	35-44y	0.84	-	1	1	1
		(0.67-1.04)				
	45-54y	0.56	-	0.66	0.61	0.71
		(0.44-0.71)		(0.55-0.78)	(0.52-0.71)	(0.52-0.97)
	55-64y	0.19	-	0.21	0.17	0.29
		(0.14-0.25)		(0.17-0.27)	(0.14-0.22)	(0.20-0.44)
	≥65y	0.02	-	0.02	0.02	0.03
		(0.01-0.04)		(0.01-0.04)	(0.01-0.04)	(0.01-0.06)
Sex (reference men)		2.19	2.81	1.96	-	-
		(1.87-2.57)	(2.51-3.14)	(1.67-2.31)		
Mid time point incidence period *		1.09	1.06	1.12	1.07	1.20
		(1.04-1.15)	(1.01-1.12)	(1.05-1.20)	(0.99-1.15)	(1.07-1.34)
Latitude degrees*		1.04	1.03	1.05	1.04	1.06
		(1.03-1.05)	(1.02-1.04)	(1.03-1.06)	(1.02-1.05)	(1.03-1.08)
Diagnostic criteria*	Poser	1	1	1	1	1
	Other than Poser’s (McAlpine) MRI data not considered	0.73	0.71	0.77	0.59	0.99
		(0.41-1.30)	(0.40-1.26)	(0.35-1.67)	(0.25-1.41)	(0.25-3.87)
	McDonald or Poser and MacDonald including MRI evidence of spread	0.68	0.43	0.94	1.06	0.76
		(0.55-0.85)	(0.35-0.53)	(0.71-1.22)	(0.80-1.41)	(0.49-1.26)

*Models including the variables indicated in results but not the survey place/time variable.

Complementary analysis revealed some discrepancies. Models for ages ≥35 years revealed the highest incidences in the UK, 1.66 (1.30-2.13), and Catania, 1.43 (1.02-2.61), with an F/M ratio of 1.96 (1.67-2.31), 1/3 lower than those seen for the younger age groups. The analysis of men aged ≥35 years at onset showed the highest, 20% (7%-34%), statistically significant increase in incidence per year, which almost tripled that seen among women of the same age, i.e., 7%. MRI-based diagnostic criteria had the highest impact on populations aged ≤35 years, 0.43 (0.35-0.53). When separate models were adjusted for age and sex, the above-mentioned incidence increased with study midpoint, i.e., ages ≥35 years in both sexes, by 12% (5%-20%) annually, together with a 5% (3%-6%) increase per degree of latitude, present across all age- and sex- or age-groups. The increase per degree of latitude was significant and rose with age and male sex, ranging from 3% for both sexes aged ≤34 years to 6% for men aged ≥35 years.

**Figure 4:**
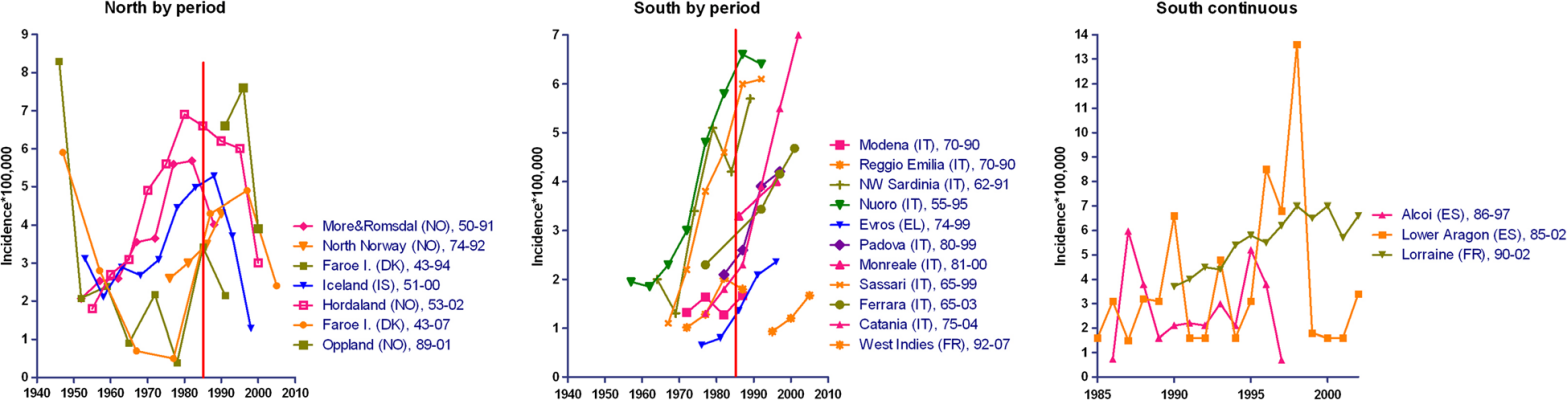
Time trends of reported MSI in the following EEA populations: (left) Nordic; (centre) Italian, Greek, and French Caribbean; (right) French mainland and Spanish.

Time series from selected surveys are depicted in Figure 4. Seven reports on Nordic, eight on Italian, and two on Greek and French Caribbean populations covered 5- to 10-year incidence figures for long periods from 1943 to 2007. Three surveys, one French and two Spanish, furnished time-series data for annual counts for the period 1985-2002. In general, MSI increased with: (a) high figures in northern and southern continental populations, reaching four to seven per 100,000 person-years but plotting different shapes, i.e., sharply decreasing in the most recent study period in northern populations (frequently attributed by authors to incomplete case-finding due to diagnostic delay) and yet increasing in Italian populations; and, (b) lower incidences in Greek and French Caribbean populations. Profiles from annual counts were difficult to assess for Alcoi and Lower Aragon (Spain) [57,58] due to unstable data, and clearly increasing for Lorraine (France) [31].

The full panorama appears to provide three different geographical and calendar-time related patterns as determined by the increasing rates, ranging from two to seven per million, and lags of approximately 10-20 calendar years. These patterns were as follows: first, northern populations generally exhibited rising incidences in the 1960s, 1970s and 1980s, with a drop in incidence in the most recent study period, and a complex, different profile with low incidence in the Faroes during the period 1960-1970 [19,49,51,55]; second, Italian populations showed heavily increasing rates from 1-2 to 6-7 per 100,000 during the 1970s, occasionally with a delay of one or more decades, such as the increase in Nuoro which was paralleled 15 years later by Catania and Lorraine, a French mainland population which, since 1990, has registered a magnitude and trend similar to that of Catania; and third, populations in Evros, Greece 1974-1999, where the starting point was the lowest among those studied, 0.66 per 100,000, and the French West Indies 1992-2007, which displayed a rise within a narrow range of 1-2.5 per 100,000 that started in Evros at the end of century and then appeared, after a 15-year delay, in the French West Indies [8,10,27,32,53].

To sum up, in post-1980 periods rates of close on 6-8 per 100,000 were seen in the majority of EEA populations, preceded by increasing and already stable trends in some Nordic (Hordaland, Oppland, Iceland), Italian (Nuoro, Sassari), and French mainland (Lorraine) populations; meanwhile, low but rising figures of 1-3 per 100,000 were still being seen at the end of the century in Greek and Caribbean populations. Where described, variations in incidence were reflected in all age-groups and both sexes. The time-related change (slope) in Nordic populations before the 1980s appeared to be replicated during the 1980s and 1990s by Italian and French populations, and at a later stage, during the 1990s and beyond, by Greek and French Caribbean populations, which solely show rising figures and shorter study periods.

## Discussion

The results of this study show that, in recent decades, MSI in EEA populations has been measured using different methods, geographically selected by residence, and has sometimes been reported by age and calendar time at “MS diagnosis”, a health-care variable that is easier to identify than that which it has replaced, i.e., “clinical onset”. Post-1985 results suggest a dynamic picture of MSI, with regular features (peak values for women aged 25-34 years, similar F/M ratios which are higher in the young age strata and youngest populations) and seven-fold variations which are better explained by local changes than by links to latitude (3-fold across 50 degrees latitude, but small if continentally considered) or time (a 2-fold increase per decade). The historical time series suggest increases, initially in the north and more recently in the south, reaching similar levels, 5-7 per 100,000 population, with figures that are still low in Greece and the French Caribbean. These results do not reveal temporal drops in incidence, except for the Faroes, age-specific bimodality or rising incidences in women linked to an increase in the F/M ratio. When it comes to data analysis and interpretation of results, limitations include: infrequent description of temporal relationships between the diagnostic process and end of case-finding periods; lack of systematic assessment of impact of health service innovations on MS diagnosis (not systematically searched for); and cultural-behavioural differences on seeking diagnosis after mild symptom onset.

**Figure 5:**
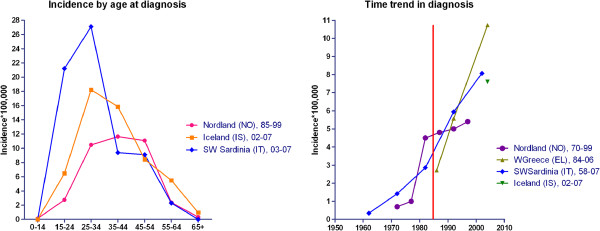
Data based on age at or year of MS diagnosis.

Selection might partly account for our results, firstly due to the choice of population by EEA neurologists conducting surveys. Secular trend analysis was mainly based on two very different but relatively homogeneous populations and medical services, i.e., Nordic and Italian. This bipolar pattern was not present in post-1985 data, in which Nordic populations were comparatively underrepresented. Interestingly, the large, sevenfold, variation is evident in both data sets, secular and post-1985, and is therefore unlikely to have been biased in the same direction by selection. Selection might have been determined by exclusion of surveys reporting age and time-point at MS diagnosis. A data plot of some surveys rejected due to incidence being calculated by age-at-diagnosis [26,30,33,46,60] indicates that three incidence features, namely, high magnitude, increase in incidence among persons aged ≥35 years and rising time trends, are more clearly revealed when seen from new diagnoses (see Figure 5) than from onsets in Figure 3. This view suggests that the increase in MS diagnoses is higher than the increase in MS onsets. The shortening of MS diagnostic delays in post-1985 surveys, likely restricted to the RRMS form [54], has frequently been described in Italian populations [28,54] but such intervals tend to be more stable in Nordic populations [59]. Diagnostic delay after symptom onset decreased from five years in the mid- or late 1980s in Norway and Italy to two years in the last decade in Spain [26,28,61]. We believe that part of the rise in MS incidence reflects improved access to neurological services at ages considerably later than age at MS onset, with it being impossible, in cases where mild first relapses are neglected, to capture patients who never reach neurological experts. The different age-specific incidence profile and results from complementary analysis showing the highest risk among women aged ≥35 years, suggest that the large UK survey [25] might contain a large proportion of women misclassified by age due to inaccurate reporting of onsets. The use of McDonald’s or theoretically more sensitive mixed criteria [62] was associated with low incidence, a paradox probably explained by the fact that such criteria were applied in survey locations and study periods when MSI was low, such as the French West Indies. The high F/M ratio in the young West Indian population and stable trend are consistent with the stable F/M ratios observed for incident patients registered in Sweden over the period 1946-2005 [63].

Time-related changes in post-1985 measurements were few, with the most relevant possibly being the rise in incidence among ≥35-year-olds. Bimodality by age at onset, as described in old surveys on black populations in the USA and recently suggested in Newcastle (Australia) for the period 1986-1996 [64], was not in evidence in our data. The explanation for both our and the Newcastle figures might conceivably lie in the capture of silent, mild MS cases by age-differential ascertainment, underlying age-differential access to neurological diagnosis bimodality in old surveys [22,65-67]. The low, extensively studied, incidences in the Faroe Islands, the modest decreasing trend in Hordaland for the 1980-1995 midpoint interval, and the occasional decline registered in Denmark [20] remain unexplained.

**Figure 6:**
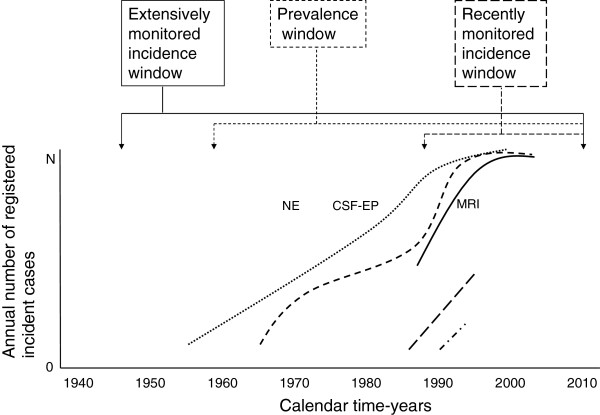
Outline of the framework for registry-based MS monitoring on different EEA populations, assuming stable annual MSI (N onsets per year) and MS diagnostic improvement secondary to synergistic effects of improved access to neurological expertise (NE), CSF oligoclonal bands, neurophysiological tests and visual evoked potentials (CSF-EP), and magnetic resonance imaging (MRI).

While substantial, several-fold variation in MSI was linked to place and only twofold variation was linked to latitude or time, such variation was not linked to more sensitive, MRI-based diagnostic criteria [33,40]. MS registration in Nordic populations, and more recently in Ferrara or Catania, shows that the magnitude of local change matches recorded international differences across longer periods and affects all age and sex groups. Improvement in diagnostic ascertainment across all ages in recent decades is evident from improved access to neurological expertise, laboratory testing and MRI, and is frequently reported in Nordic and Italian studies. The fact that earlier low incidence persists being low after prolonged survey updates in Ferrara, Catania and Hordaland, may suggest that diagnostic improvement basically affects clinically recent and mildest MS forms. Such ubiquitous factor might explain secular and post-1985 time trends in the EEA. This interpretation is difficult to reconcile with two views held by local researchers claiming a true increase in MSI: first, that suggested by Italian and mainland-based French neuroscientists, whereby the increase is said to be attributable to the biological impact of changes in women’s life-style [10,23,31,44]; second, that relatively restricted to returning continental immigration, reported in the French West Indies as a lagged phenomenon imported from a higher-risk environment [8,68]. Major reasons for rejecting such interpretations or modulating them in favour of better detection in mild cases are: (1) that the increase in MSI among women or older women is, if anything, restricted to single surveys (not seen here), and that changes in EU women's lifestyles (e.g., occupational) were generalised and predated studies on Scandinavian populations not showing such an increase, (http://www.ilo.org); (2) that rising MSI in the French West Indies pertains to particularly severe forms [8,69], thus potentially representing the tip of the iceberg already uncovered in the majority of other surveyed EEA populations, and (3) rising MSI is seen in men. Disregarding potential effects of D Vitamin dietary or childhood hygienic changes, race and latitude, we would prefer to propose that increased population concern about MS symptoms, referral and access to neurological expertise and, recently, to technology acting on stable MSIs might account for the entire EEA pattern, particularly due to the increasing proportion of women active in non-domestic occupational work among those of working age in EEA-surveyed populations. The geographically and time-related decreasing differences in MSI in the EEA by asymptotic trend towards an incidence of 5-7 per 100,000, may suggest that MS appears to behave invariantly with respect to time and space at a stage characterised by improvements in diagnosis and treatment, and by the loss of the natural history due to the widespread use of immune-modulatory treatments that are partly effective and not free of side-effects [70,71]. MS diagnostic functions at EEA might mirror the parallel logarithmic or sigmoid functions outlined in Figure 6. Such pattern defines a framework that is inevitably present in different approaches (surveys, registries, monitoring systems), potentially providing what are apparently diverging but are, in reality, consistent results on different patients. On combining worldwide data from 38 incidence periods reported in 1966-2007, i.e. the global longitudinal approach, Alonso and Hernan [25] describe increasing time-trends for incidence and F/M ratios attenuated after 1980, findings that are consistent with ours. Register-based, calendar-time-limited incident approaches (see the post-1985 window in Figure 6) and prevalence approaches [72] accumulate an increasing proportion of frequently treated cases detected in high-incidence settings.

Given the suggested difficulties found in measuring MSI, it would appear that the proposal of a pilot surveillance system active under suspect alarm conditions, similar for instance to those designed and tested for Guillain-Barré syndrome [73], may not be opportune for a number of reasons (lack of reported outbreaks or an expected preventive response, problems managing suspected cases), at least until MSI levels exceed those described for Nordic populations or new aetiological agents are identified. Instead, some of the existing monitoring alternatives appear to be reasonable, subject to being population- rather than hospital-based. Fortunately, there is long experience of monitoring EEA populations.

## Conclusions

We conclude that the reported rising incidence of MS in the EEA might be attributable to improved ascertainment and that population-based MS monitoring by selected centres may be useful.

## Competing interest

The authors declare that there are no conflicts of interest. Enrique Alcalde-Cabero’s work was partly funded by the above-mentioned grant from Biogen Idec.

## Authors’ contributions

E Alcalde-Cabero contributed to the design of the methods and protocols, refined and managed the study database, and conducted preliminary tabulations and statistical analyses; J Almazán-Isla supervised data collection and data entry; J. de Pedro-Cuesta conceived the study, coordinated the research team, and collaborated with E Alcalde on the draft of the first manuscript. All authors contributed by commenting on the manuscript.

## Pre-publication history

The pre-publication history for this paper can be accessed here:

http://www.biomedcentral.com/1471-2377/13/58/prepub
